# The Effects of Sweet Foods on the Pharmacokinetics of Glycyrrhizic Acid by icELISA

**DOI:** 10.3390/molecules22030498

**Published:** 2017-03-21

**Authors:** Bingqian Jiang, Huihua Qu, Hui Kong, Yue Zhang, Shuchen Liu, Jinjun Cheng, Xin Yan, Yan Zhao

**Affiliations:** 1 School of Basic Medical Sciences, Beijing University of Chinese Medicine, 11 Beisanhuandong Road, Chaoyang District, Beijing 100029, China; 20130941016@bucm.edu.cn (B.J.); doris7629@126.com (H.K.); jinzy0423@163.com (Y.Z.); liushuchensun@163.com (S.L.); carlosjjcheng@163.com (J.C.); 2 Institute of Traditional Chinese Medicine, Beijing University of Chinese Medicine, 11 Beisanhuandong Road, Chaoyang District, Beijing 100029, China; quhuihuadr@163.com; 3 School of Chinese Materia Medica, Beijing University of Chinese Medicine, 11 Beisanhuandong Road, Chaoyang District, Beijing 100029, China; 20150931805@bucm.edu.cn

**Keywords:** glycyrrhizic acid, icELISA, pharmacokinetics, sweet foods

## Abstract

The effect of sweet foods, such as honey, was investigated from the perspective of pharmacokinetics on the absorption of glycyrrhizic acid (GA). Due to the unique properties of indirect competitive enzyme-linked immunosorbent assay (icELISA), namely, its: specificity, sensitivity, repeatability, simple pretreatment of samples, fast and simple operation, and because it is economic and non-polluting, it has received increased attention. In this study, we used the advantages of this method to see how honey affected the pharmacokinetics of GA. The effects of honey on the pharmacokinetics of GA by ELISA were investigated for the first time. The results indicate that honey can postpone the peak concentration of GA in mouse blood, and this effect correlates well with fructose. As a representative of sweet foods, the result provides the valuable information that honey, or fructose, may act as sustained-releasing drugs in clinical scenarios; and that sweet foods may have some influences on drugs when taken together.

## 1. Introduction

Honey is widely used in our daily life as a nutrient and is representative of sweet foods. It is produced by using the juice secreted by plant glands after bees have gathered nectar. There are over 300 types of floral honey available in the market worldwide. The color, flavor, and mineral and vitamin content of honey depend on the flower from which bees gather the nectar [1]. However, usually, honey mainly contains glucose and fructose, which make up 65%–80% of the total weight of honey. In addition, sucrose makes up less than 5% of the total weight of honey.

Zhi Gancao is a famous Chinese herb produced by processing licorice with honey. According to the theory of traditional Chinese medicine, the sweet taste has the effect of slowing down the person who experiences it. However, no elucidation of this theory has been done until today. It is generally accepted that raw licorice is not as potent as processed licorice on many diseases compared to other Chinese herbs in clinical applications. The traditional Chinese medicine composition is complex, and research on the interaction of effective components in vivo can provide experimental support for the rational use of traditional Chinese medicine. Many reports show that, after processing, the effect of the change of drug action was proved to be reasonable [2].

Glycyrrhizic Acid (GA), also named Glycyrrhizin, is the highest content of licorice and has broad and robust biological activities, e.g., anti-inflammatory, antiviral, antibacterial, anti-allergy (anti complement activity), protecting the membrane of the liver cell, enhancing immune function, inducing interfero, etc. [3,4,5,6]. Relative mechanism research indicates that GA could inhibit 11β-HSD1 and 11β-HSD2, and thus downregulate the level of inflammatory factors such as IL-6, resulting in it playing an anti-inflammatory, antiviral and antibacterial role [7,8].

A recent method commonly used or recommended in the practice of domestic traditional Chinese medicine (TCM) quality control is high performance liquid chromatography (HPLC). It has been included in the national pharmacopoeia [9,10,11,12,13]. However, the sample pretreatment processes, such as extraction and purification of active ingredients by HPLC is rather complex. Moreover, the HPLC method is time-consuming, requires the use of organic solvents, depends on large cost of instruments and equipment, and has high professional technical requirements for the operator. In contrast, enzyme linked immunosorbent assay (ELISA) is established by using a monoclonal antibody, its advantages include: specificity, sensitivity, repeatability, simple pretreatment of sample, fast and simple operation, economic and no-polluting, etc. Therefore, it has received more and more attention [14,15,16,17,18]. It is capable of analyzing large number of samples simultaneously, especially in cases where there is a very low content of the active ingredient. The technique for the analysis of active components of medicine will be important to complementary technologies and in establishing effective means.

In this paper, an indirect competitive enzyme linked assay (icELISA) was established for determination of GA to study the pharmacokinetics activities of GA in mice plasma. The effect of honey on pharmacokinetics activities of GA in mice plasma after being administered with licorice extract and GA were studied. Furthermore, we attempted to look at how the most correlated components (glucose, fructose and sucrose) contributed to the effect which honey plays in pharmacokinetics activities of GA in mice plasma.

## 2. Results and Discussion

### 2.1. icELISA Determination of GA

**Figure 1:**
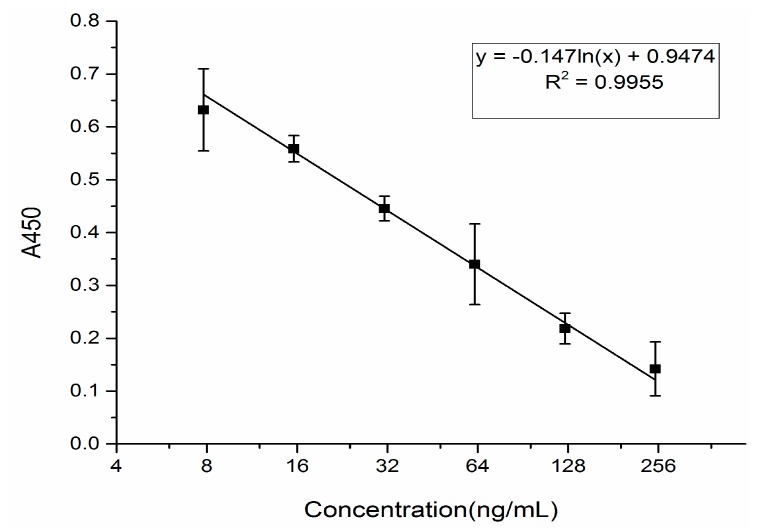
The concentration of glycyrrhizic acid (GA).

The icELISA for the determination of GA has been previously established in our lab [19]. In this study, the determination of GA in mouse blood by icELISA was assayed. The standard curves of GA for the measured range was created as follows (Figure 1), where the regression equation of GA was y = −0.14ln(x) + 0.947, R = 0.995. The linear range of GA was from 7.8 to 250 ng/mL, respectively. The value of IC_50_ for GA was 24.36 ng/mL.

As the results indicate, the icELISA method for GA had good sensitivity and specificity. The ELISA method has a strong capacity for measurement without expensive instruments and complex pretreatment. Furthermore, the ELISA assay requires low quantities of sample material, for example, 5 µL of serum was sufficient for the determination of GA in mice in our study.

### 2.2. The Effects of Honey on Pharmacokinetic Parameters of GA after Given Licorice Extract (LE) with or without Honey in Mouse Blood

**Figure 2:**
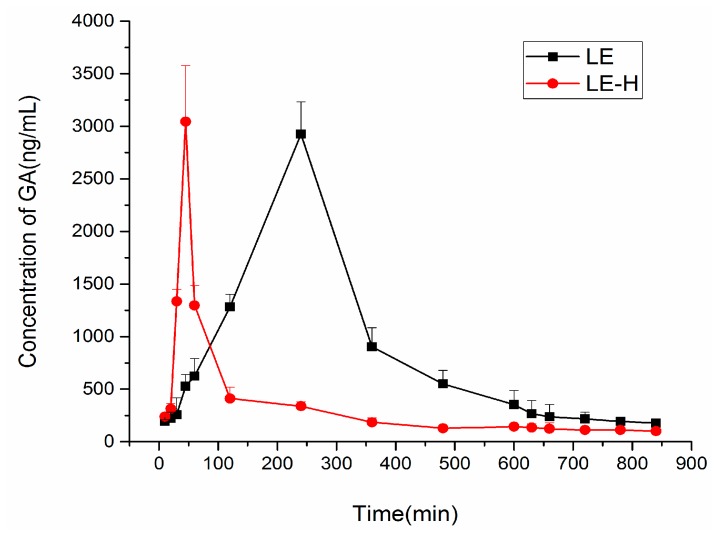
Effects of honey on the blood concentration time curve of glycyrrhizic acid (GA).

The icELISA method described above was applied to the analysis of blood samples obtained from mice administered intragastrically. After licorice extract (LE) (with and without honey) was administered, respectively, blood samples were collected at different time points to detect the blood concentration time curve of GA. The mean GA concentration-time profile is presented in Figure 2, where GA showed a peak of maximum blood concentration in mice after the administration of a single licorice extract at 45 min, while the peak of maximum blood concentration in mice after being given licorice extract with honey was at 240 min.

The pharmacokinetic parameters were calculated using Kinetica 5.0 pharmacokinetics software (Thermo Fisher Scientific, Waltham, MA, USA). The pharmacokinetic parameters obtained from the blood samples of mice following the oral administration of LE are shown in Table 1.

### 2.3. The Effects of Honey on Pharmacokinetic Parameters of GA after Given GA Solutions in Mouse Blood

**Figure 3:**
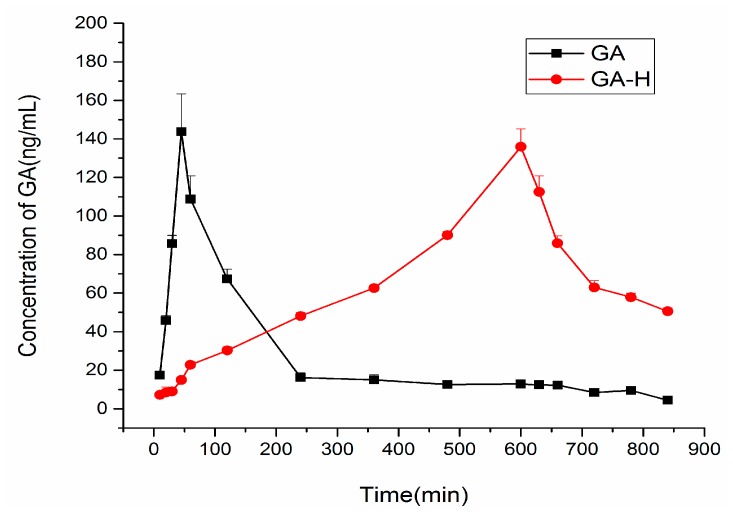
Effects of honey on the blood concentration time curve of glycyrrhizic acid (GA).

After glycyrrhizic acid (with and without honey) was administered, blood samples were collected at different time points to detect the time curve of GA concentration in blood. The concentration time curves of the GA and the combination of GA and honey are shown in Figure 3. As the figure shows, the addition of honey to GA when compared to GA administered alone, slows down the absorption and elimination process of GA, and this becomes even slower when compared with licorice extracts with and without honey. The peak concentration of GA after the administration of a GA solution was at 45 min, while it was 600 min when co-administered with honey. Therefore, as the addition of honey had a significant influence on the metabolism and excretion of GA in mice, honey may delay the GA peak concentration.

The pharmacokinetic parameters were calculated using Kinetica 5.0 pharmacokinetics software. The pharmacokinetic parameters obtained from the blood samples of mice following oral administration of GA solutions are shown in Table 2.

### 2.4. Effect of Important Components of Honey on Pharmacokinetics of GA in Mice

**Figure 4:**
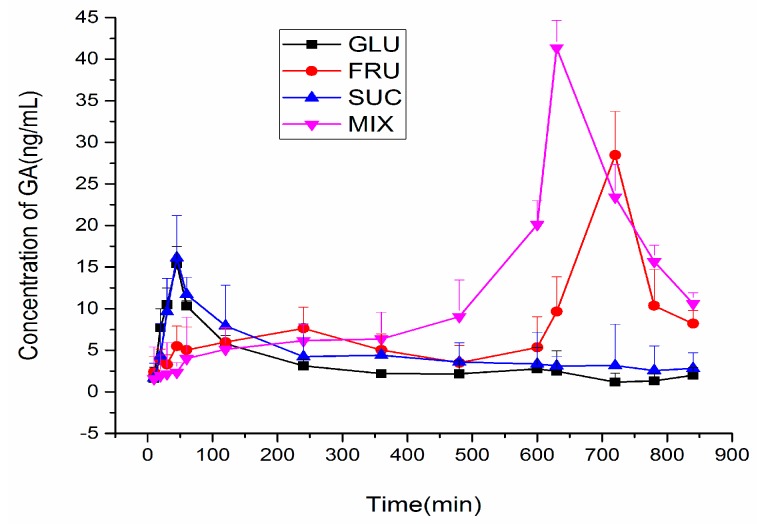
Effects of honey on the blood concentration time curve of glycyrrhizic acid (GA).

In this experiment, three of the most important components in honey (glucose, fructose, sucrose) were investigated to further explore the material basis in honey on the effect of delaying the peak concentration of GA in mouse blood. After a corresponding drug was administered separately, blood samples were collected at different time points to detect the blood concentration time curve of GA in different groups. The pharmacokinetic parameters were calculated using Kinetica 5.0 pharmacokinetics software. The concentrations of GA were detected, and the mean concentrations were calculated and plotted. Figure 4 reflects how the concentrations of GA vary against time across the different groups. On the basis of this assay, the Cmax, Tmax, AUC0-t and MRT were calculated (As shown in Table 3).

As the Figure 4 and Table 3 reflects, the time to peak concentration of GA in Glucose (GLU)/Sucrose (SUC) group was both at 45 min, while the time to peak in the Fructose (FUC) group and Mix Solution of GLU, SUC and FUC (MIX)group were 720 min and 630 min, respectively. The time to peak in the FUC group and MIX group were greatly postponed, which was similar to the blood concentration time curves of GA when adding honey to GA. In these four groups, the time to peak in the FUC group is the latest one, while the peak intensity in the MIX group was the highest. The concentrations of GA variation versus time in different groups showed significant differences, with the exception that no significant changes were found in the GLU and SUC groups. The FRU group was the most similar to the MIX group, which suggests that fructose plays the most important role in honey in delaying the peak concentration of GA in mice plasma.

A lot of related literatures have been reported about the detection of GA [20,21,22,23,24,25,26,27], but the direct detection of GA in the blood of mice is yet to be reported. It is common sense that drugs should be eaten 30 min before or after a meal. Whether the sweet foods in a meal will influence the absorption of licorice or not is unknown. Taking advantage of this method, we attempted to study the effects of honey on the pharmacokinetics of GA in mice blood from intragastric administration with licorice extract to GA solution.

From the result of GA determination by ELISA, there were significant differences in the GA concentrations at peak times between the groups of licorice extract with and without honey in mice, suggesting that the administration of licorice extract with and without honey can lead to varied plasma concentrations of GA, and the honey plays the vital role in these differences.

Specifically, comparing the group administered with only licorice extract with the group co-administrated with licorice extract and honey, the absorption and elimination process of GA becomes slow in the co-administration group, which means honey added to licorice extract may have some influence on the metabolism and excretion of GA in mice. The differences were found to be similar in groups administered with GA solution and GA with honey.

It is not easy to change GA into a polymer, and it is always seen as a monomer in the gastrointestinal tract, so it is easy to digest. As related literatures have reported, the concentration of GA is inclined to increase to the maximum concentration, but it also rapidly dropped [28,29]. This is consistent with the result showed above for the pharmacokinetics of GA when administered with GA solution alone. However, we noticed that the honey has a robust effect on the pharmacokinetics of GA; it makes the absorption and metabolism process of GA in mice blood become much slower. Furthermore, three components (glucose, fructose and sucrose) of honey were observed for their effect on GA pharmacokinetic processes. The results showed the honey exhibited a moderate activity, which correlates well with its fructose composition.

The results indicate that sweet food may influence the absorption of licorice, especially when eating food that contains a high amount of fructose. Moreover, a high intake of fructose is easy to achieve in our daily life, because fructose exists in high quantities in apples and pears, and tropical fruits like mango, and citrus, peach, plum, apricot, etc., contain even more sugar, half of which will be converted to fructose after catabolism.

Both the studies underline the impacts of eating sweet food or fructose together with licorice, because sweet food, like honey, can result in postponing the time when licorice will work, which means it could act as a sustained-releasing drug, which is of great importance in clinical scenarios. It is also important that people are made aware of the potential effects of eating licorice in sweet fruit juice drinks. There are various kinds of such drinks in the market, and these juice drinks have abundant levels of fructose.

From the perspective of medicine, the mystery of processing is revealed to some extent in terms of the effect that drug interaction has on the curative effect. This is the internal mechanism of the therapeutic effects exerted by traditional Chinese Medicine.

As glycyrrhetinic acid (GE) is the metabolite of GA and is the active principle responsible for most of the positive and negative effects of licorice, we will further focus on the GE measurement in our following studies.

## 3. Materials and Methods

### 3.1. Reagents and Instruments

GA (purity: 95.2% and batch number: 110715-200815), was purchased from the National Institute for Food and Drug Control (NIFDC, Beijing, China). Licorice in Chinese herbs was purchased from the China Resources Sanjiu Medical & Pharmaceutical Co., Ltd. (Beijing, China). Honey was purchased from the Shanghai HuJiao Bee Industry Association Co., Ltd. (Shanghai, China). Glucose, sucrose and fructose were purchased from the Beijing Chemical Reagent Company (Beijing, China).

Immunoplates (96-well) were purchased from Corning Incorporated (Suzano, NY, USA). A coating antigen GA-BSA was produced in our laboratory. Skim milk powder for laboratory use only was purchased from OXOID Corporate (Beijing, China). Goat anti-mouse immunoglobulin conjugated to horseradish peroxidase (GaMIgG-HRP; whole molecule) was purchased from GE Healthcare (Chicago, IL, USA). Furthermore, 3,3,5,5-Tetramethylbenzidine (TMB) was purchased from Sigma-Aldrich, Co. (Beijing, China).

All other commercial chemicals used in this study were of analytical reagent grade and were obtained from Sinopharm Chemical Reagents Beijing Co., Ltd. (Beijing, China).

A spectrophotometric microtiter reader (ELx 800) (BioTek Company, Winooski, VT, USA) was used for the absorbance measurements.

### 3.2. The icELISA for the Determination of GA

The icELISA for the determination of GA in mouse blood was established. Assay validation, including evaluations of standard curves, precision, stability, accuracy, and recovery of GA in the blood were also performed.

The icELISA procedures were as follows: Each well of a 96-well microplate was coated with 100 μL of the coating antigen diluted with Carbonate Buffer Solution (CBS). Plates were incubated for 1 h at 37 °C, and then washed three times with PBS-T. A blocking buffer (200 μL) was added to each well, and the plates were sealed and blocked for 1 h at 37 °C. Monoclonal Antibodies (MAbs) diluted with Phosphate-Buffer Saline (PBS) (50 µL) were added to each well, followed by the addition of an equal volume of the blood samples diluted with PBS. After incubation for 1 h at 37 °C, the plates were washed three times. Next, 100 μL of goat anti-mouse IgG diluted 1:10,000 in PBS was added to each well and the plates were sealed and incubated for 0.5 h at 37 °C. After three washes, 100 μL of TMB substrate solution was added. The plates were sealed, protected from light and incubated at 37 °C. The reaction was stopped after 15 min by adding 50 μL of stop solution and the plates were read at 450 nm. Blank blood and PBS were used as the negative control and blank, respectively.

### 3.3. Animals

Male Kunming mice, weighing 30 ± 2 g, were purchased from the Beijing Vital River Laboratory Animal Technology Co., Ltd. (Beijing, China). Animals were housed in an environmentally controlled breeding room for 1 week prior to the start of the experiments and provided with standard laboratory food and water.

### 3.4. Establishment of Detection Method of ELISA of GA

The icELISA protocol used to measure the concentrations of GA was previously established. Assay validation, including evaluations of standard curves, precision, stability, accuracy, and recovery of GA in the blood and PBS were also performed.

The icELISA procedures were as follows: Each well of a 96-well microplate was coated with 100 μL of coating antigen diluted with CBS. Plates were incubated for 1 h at 37 °C, and then washed three times with PBS-T. A blocking buffer (200 μL) was added to each well, and the plates were sealed and blocked for 1 h at 37 °C. MAbs diluted with PBS (50 µL) was added to each well, followed by the addition of equal volume of the blood samples diluted with PBS. After incubation for 1 h at 37 °C, the plates were washed three times. Next, 100 μL of goat anti-mouse IgG diluted 1:10,000 in PBS was added to each well and the plates were sealed and incubated for 0.5 h at 37 °C. After three washes, 100 μL of TMB substrate solution was added. The plates were sealed, protected from light and incubated at 37 °C. The reaction was stopped after 15 min by adding 50 μL of stop solution and the plates were read at 450 nm. Blank blood and PBS were used as the negative control and blank, respectively.

### 3.5. Pharmacokinetics Experiment

This study was performed as per the Guidelines for the Care and Use of Laboratory Animals and was approved by the Committee of Ethics of Animal Experimentation of the Beijing University of Chinese Medicine. Mice were fasted overnight and for an additional 12 h prior to drug administration.

Three experiments are included in this paper. In Test 1, 20 male Kunming mice were randomly divided into two groups of 10 mice each, with one group orally administered with a water extract of glycyrrhiza (GA concentration = 638 µg/mL), and the other being given a mixture of honey and licorice extract (the amount of honey calculated according to the mass ratio used for processing licorice using honey as the adjuvants, in which the mass ratio of honey compared with the licorice extract is 4:1).

After administration at 10 min, 20 min, 30 min, 45 min, 60 min, 120 min, 240 min, 360 min, 480 min, 600 min, 630 min, 660 min, 720 min, 780 min, and 840 min, 5 µL tail blood was collected via the tail vein with a quantitative capillary (pre-dipped with heparin sodium) and the blood was diluted 10-fold with PBS immediately and preserved at −20 °C. In Test 2, the groups were administered with a GA solution and a GA solution with honey, and the other steps remain the same as Test 1.

In Test 3, the two groups were administered with a water solution of glucose, fructose, sucrose, and a mixed solution of glucose, fructose and sucrose, respectively. It is worth noting that the concentration ratio of the three components of honey, as per the ratio in honey, was 28:40:5 (based on a previous report on the content of these three components in honey) [30,31,32].

### 3.6. Blood Sample Treatment

After the blood samples were thawed to room temperature, 5 µL samples were diluted 10 times with PBS for measurement.

### 3.7. Data Analysis

The pharmacokinetic parameters such as the mean maximum blood concentration (Cmax), time of maximum concentration (Tmax), terminal elimination half-life, area under the curve (AUC0-t), and mean residence time (MRT) were calculated using Kinetica 5.0 software (Thermo Fisher Scientific) in the non-compartment model analysis method, and the pharmacokinetic parameters of the two-tailed *t*-test were statistically analyzed, with a value of *p* < 0.05 being statistically significant.

## 4. Conclusions

Honey may have a delaying effect on the absorption of GA, and this is mainly caused by fructose, which is a very important component in honey and is present in high levels in everyday foods. This paper shows the delaying effects of honey, which is valuable in reminding us of the probable effects when taking drugs with sweet foods and may act in the sustained-release process of drugs in clinical scenarios. Furthermore, it provides research potential in the processing area of using honey as adjuvants.

## Figures and Tables

**Table 1:** Pharmacokinetic parameters of glycyrrhizic acid after administration of licorice extract with and without honey (mean ± SD, *n* = 8).

GA	Licorice Extract	Licorice Extract with Honey
Cmax (mg·L^−1^)	3045.22 ± 116.92	2025.59 ± 84.97
Tmax (min)	45.00 ± 8.90	240.00 ± 138.52
AUC0–840 (mg·min·L^−1^)	17,385.33 ± 1567.36	48,605.87 ± 7797.37
AUCto-t	336,236 ± 10,450.14	842,884 ± 34,613.27
MRT (min)	233.50 ± 106.35	301.54 ± 266.50

**Table 2:** Pharmacokinetic parameters of GA after administration of GA solution with and without honey (mean ± SD, *n* = 8).

	GA	GA with Honey
Cmax (mg·L^−1^)	143.81 ± 19.34	88.53 ± 8.60
Tmax (min)	45.00 ± 25.20	600.00 ± 92.53
AUC0–840 (mg·min·L^−1^)	12,575.80 ± 8482.22	3619.53 ± 3835.65
AUCto-t	22,791.4 ± 3180.61	81,918.3 ± 6715.43
MRT (min)	235.02 ± 86.38	500.02 ± 169.70

**Table 3:** Pharmacokinetic parameters of GA after administration of licorice extract with or without honey (mean ± SD, *n* = 8).

GA	GLU	FRU	SUC	MIX
Cmax (mg·L^−1^)	16.56 ± 7.64	28.46 ± 12.60	16.12 ± 9.82	41.36 ± 23.29
Tmax (min)	45.00 ± 12.20	720.00 ± 191.83	45.00 ±25.80	630.00 ± 126.74
AUC0–720 (mg·min·L^−1^)	200.13 ± 155.72	480.22 ± 289.18	279.90 ±121.76	728.11 ± 405.39
AUCto-t	6801.75 ± 530.56	7514.27± 925.43	7274.90 ± 738.35	11,797.50 ± 1285.33
MRT (min)	849.77 ± 316.19	564.451 ± 221.70	1112.67 ± 793.85	620.61 ± 285.93
